# Supplementary material for: Longitudinal Association of Maternal Pre-Pregnancy BMI and Third-Trimester Glycemia with Early Life Growth of Offspring: A Prospective Study among GDM-Negative Pregnant Women
Source: Nutrients. 2021 Nov 7;13(11):3971. doi: 10.3390/nu13113971 (PMC8619788; doi:10.3390/nu13113971)
Supplement: Supplementary file 1 [file nutrients-13-03971-s001.zip › nutrients-1395199-supplementary.pdf]

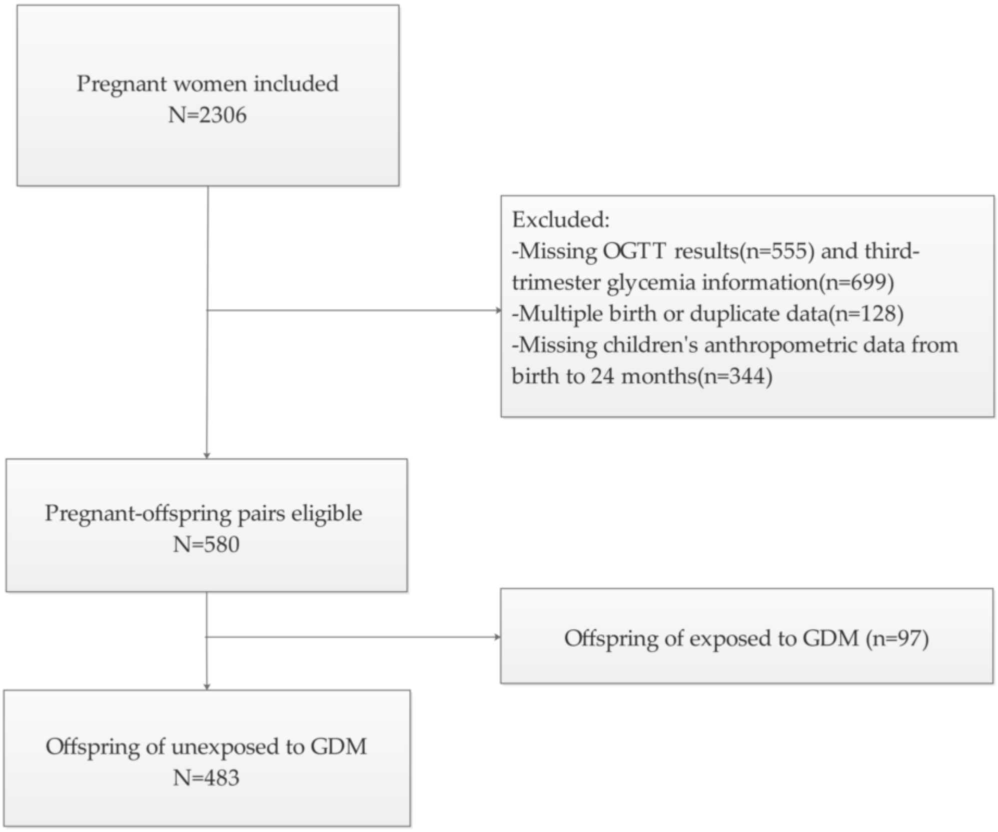

Figure S1. Study participants flow chart.

Table S1. Comparisons of main characteristics of pregnant women in different groups.

Variable	Group 1: pregnant women included in the analysis(n=2306)	Group 2: pregnant women recruited in the cohort(n=483)	*P*-value
**Maternal characteristics**			
Age at delivery, mean±SD, (years)	28.0 ± 3.9	28.0 ± 4.0	0.862
Gestational weight gain, mean±SD, (Kg)	15.5 ± 5.4	15.6 ± 5.1	0.836
Gestational hypertension	208 (10.2)	73(15.1)	0.141
Prepregnancy BMI, mean±SD, (kg/m2)	21.3 ± 2.9	20.9 ± 2.7	0.524
Prepregnancy BMI category			0.297
18.5~23.9	1401 (68.7)	317 (65.6)	
<18.5	321 (15.7)	76 (15.7)	
≥24.0	317 (15.5)	90 (18.7)	
Education level>9 (years)	1632(70.8)	401(83.0)	0.184
Family income ≤ ¥ 200 thousand (RMB)	1656 (81.2)	398 (82.4)	0.918
Parity- nulliparous	1142 (56.0)	287 (59.4)	0.063
**Offspring characteristics**			
Gestational age at delivery, mean±SD, (weeks)	39.3 ± 1.3	39.3 ± 1.2	0.183
Gender-Male	998 (48.9)	240 (49.7)	0.365
Delivery method-cesarean section	1053 (51.6)	235 (48.7)	0.061
Birth weight, mean±SD, (kg)	3.4 ± 0.4	3.4 ± 0.4	0.851

Data are shown as n (%) unless otherwise indicated. Based on $\chi 2$ test, with Fisher exact test used for variables with any cell count < 10, or Kruskal-Wallis test for continuous variables, $P < 0.05$.
